# Spatial and environmental variables structure sponge symbiont communities

**DOI:** 10.1111/mec.16631

**Published:** 2022-08-10

**Authors:** Daniel F. R. Cleary, Ana R. M. Polónia, Thomas Swierts, Francisco J. R. C. Coelho, Nicole J. de Voogd, Newton C. M. Gomes

**Affiliations:** ^1^ CESAM ‐ Centre for Environmental and Marine Studies, Department of Biology University of Aveiro Aveiro Portugal; ^2^ Marine Biodiversity, Naturalis Biodiversity Center Leiden The Netherlands; ^3^ Institute of Environmental Sciences (CML) Leiden University Leiden The Netherlands

**Keywords:** distance, HMA sponges, LMA sponges, prokaryotic communities, seawater, sediment

## Abstract

Understanding the maintenance and origin of beta diversity is a central topic in ecology. However, the factors that drive diversity patterns and underlying processes remain unclear, particularly for host‐prokaryotic associations. Here, beta diversity patterns were studied in five prokaryotic biotopes, namely, two high microbial abundance (HMA) sponge taxa (*Xestospongia* spp. and *Hyrtios erectus*), one low microbial abundance (LMA) sponge taxon (*Stylissa carteri*), sediment and seawater sampled across thousands of kilometres. Using multiple regression on distance matrices (MRM), spatial (geographic distance) and environmental (sea surface temperature and chlorophyll α concentrations) variables proved significant predictors of beta diversity in all five biotopes and together explained from 54% to 82% of variation in dissimilarity of both HMA species, 27% to 43% of variation in sediment and seawater, but only 20% of variation of the LMA *S. carteri*. Variance partitioning was subsequently used to partition the variation into purely spatial, purely environmental and spatially‐structured environmental components. The amount of variation in dissimilarity explained by the purely spatial component was lowest for *S. carteri* at 11% and highest for *H. erectus* at 55%. The purely environmental component, in turn, only explained from 0.15% to 2.83% of variation in all biotopes. In addition to spatial and environmental variables, a matrix of genetic differences between pairs of sponge individuals also proved a significant predictor of variation in prokaryotic dissimilarity of the *Xestospongia* species complex. We discuss the implications of these results for the HMA‐LMA dichotomy and compare the MRM results with results obtained using constrained ordination and zeta diversity.

## 1 INTRODUCTION

Beta diversity is an important concept for understanding how ecosystems function and can be studied by assessing the change in community dissimilarity in relation to geographic distance (distance‐dependence; Ellingsen, 2002; Legendre et al., 2005; Nekola & White, 1999). Initially studied in communities of multicellular organisms (Becking et al., 2006; Cleary et al., 2004, 2005, 2008, 2016; Condit et al., 2002; de Voogd et al., 2006, 2009; Nekola & White, 1999; Preston, 1960; Tuomisto et al., 2003), a large number of studies have also highlighted the existence of distance‐dependence in miccrobial communities (Chen et al., 2020; Clark et al., 2021; Gao et al., 2019; Green et al., 2004; Hanson et al., 2012; Landesman et al., 2014; Lear et al., 2014; Martiny et al., 2006, 2011; Meyer et al., 2018; Papke et al., 2003; Whitaker et al., 2003; Wu et al., 2018). Studying distance‐dependence in microbial communities is of particular interest because microbes were assumed to be ubiquitous (“Everything is everywhere, but the environment selects”; Baas Becking, 1934). Their very large population sizes and seeming ease of dispersal would also appear to overcome dispersal limitation. Some studies have indeed failed to find an association between distance and community dissimilarity (cited in Clark et al., 2021). In addition to living in soil, sediment or water, microbes can also associate with multicellular host organisms. These microbial communities can form stable, host‐specific communities, which influence host fitness and perform crucial functions (Thompson et al., 2015).

In the present study, we sampled three host sponge taxa, sediment and seawater. Besides taxonomic distinctions, sponges can also be distinguished by the density of their microbial communities (Hentschel et al., 2002, 2003; Reiswig, 1981; Vacelet & Donadey, 1977). High microbial abundance (HMA) sponge species, also known as ‘bacterial sponges’, tend to house dense and diverse microbial communities in contrast to low microbial abundance (LMA) sponge species, which tend to house less dense and diverse microbial communities although the differences are not always evident. In addition to microbial density, these groups also differ morphologically with HMA sponges generally having greater mesohyl density while LMA sponges have larger choanocyte chambers and higher pumping rates.

Previous studies have shown that both LMA and HMA sponges housed prokaryotic communities, which were distinct from those found in sediment and seawater; prokaryotic communities of LMA sponges have, however, been shown to be more similar to prokaryotic communities found in seawater (Cleary et al., 2015, 2018, 2019, 2020; Hentschel et al., 2002, 2003; Moitinho‐Silva et al., 2017). HMA sponges, in contrast, housed highly distinct prokaryotic communities characterized by relatively high evenness (compared to LMA sponges) and high abundances of selected phyla including Chloroflexi and Poribacteria, which were relatively rare components of seawater and most LMA sponges (Bayer et al., 2014; Cleary et al., 2015, 2018; de Voogd et al., 2015, 2019; Moitinho‐Silva et al., 2017; Polónia, Cleary, Freitas, et al., 2015).

A large number of studies have found sponge‐associated bacterial communities to exhibit pronounced spatial stability including studies with samples taken thousands of kilometres apart (Cárdenas et al., 2018; Hentschel et al., 2002; Hill et al., 2006; Pita et al., 2013; Reveillaud et al., 2014; Taylor et al., 2005; Webster et al., 2004). Pita et al. (2013), for example, found no significant relationship between geographical distance and similarity across a scale of hundreds of kilometres for the HMA sponge *Ircinia* sp. in the Mediterranean Sea. More recent studies have, however, identified significant differences in prokaryotic composition between geographically separated populations of several HMA and LMA sponge species (Díez‐Vives et al., 2020; Ferreira et al., 2020; Griffiths et al., 2019; Marino et al., 2017; Swierts et al., 2018; Taylor et al., 2005). These contrasting results would appear to warrant a more formal testing of the impact of geographic distance on sponge‐associated prokaryotic dissimilarity.

Considerable debate has focused on the underlying mechanisms responsible for observed distance‐dependence patterns (Clark et al., 2021). Communities of organisms, for example, may respond to local environmental conditions or alternatively distance‐dependence may be the result of dispersal limitation or a combination of both processes (Clark et al., 2021; Spencer et al., 2002). Dispersal limitation may arise due to the spatial configuration of habitat, whereby the habitat matrix plays an important role, and/or because of intrinsic differences in dispersal ability among groups of species. For example, the habitat configuration may promote dispersal via stepping stones or networks. Stepping stones are suitable habitat patches across a species’ distributional range, which can enable a species to “island‐hop” from one suitable habitat patch to the other. A habitat matrix with a greater density of stepping stones should, in theory, enable greater dispersal rates.

Other factors, which can influence community composition are historical contingency and priority effects (Hanson et al., 2012). The relationship between the spatial configuration of habitats and the habitat matrix, and intrinsic dispersal ability, furthermore, influences the duration of historical effects on ecosystems (Nekola & White, 1999). Historical contingency entails that the observed composition is at least partially constrained by historical events. Vertical transmission of the microbiome, for example, from parent to offspring may differ substantially among individual organisms, yet play an important role in shaping the microbiome throughout the life of the organism. Priority effects also play a role in historical contingency and entail the impact that a given species or sets of species have on community development depending on their time of arrival. Early arriving species can, for example, hinder subsequent colonization by other species, either actively or by modifying the available resources. Alternatively, initial colonizers may facilitate subsequent colonization of other species.

There are a number of hypotheses with respect to the maintenance and origin of beta diversity. These include (1) composition is uniform, which emphasizes biotic interactions and dominance of a limited set of competitively dominant species; (2) composition is spatially autocorrelated, which emphasizes dispersal limitation and assumes that species are demographically and competitively equal; and (3) composition is determined by local environmental conditions (Legendre et al., 2005). In the present study, we assessed the degree to which spatial (geographic distance) and environmental factors (sea surface temperature [SST] and chlorophyll α [Chlα] concentrations) structured prokaryotic communities in three sponge taxa in addition to sediment and seawater. In previous studies, we showed that satellite‐derived SST and/or Chlα concentrations were significant predictors of variation in the composition of a range of multicellular organisms including corals, fishes, ascidians and sponges in Indonesian coral reef systems (Cleary et al., 2016; Polónia, Cleary, de Voogd, et al., 2015). Temperature and Chlα concentrations have also been identified as significant predictors in the variation of zooplankton and bacterial species and communities in several marine environments (Chow et al., 2013; Huq et al., 1984; Johnson et al., 2012; Julie et al., 2010; Stauder et al., 2010; Sun et al., 2021). In addition to the above, we also tested to what extent a matrix of genetic differences between pairs of sponge individuals was able to explain variation in prokaryotic dissimilarity of the giant barrel sponge (*Xestospongia*) species complex.

## 2 MATERIALS AND METHODS

### 2.1 Sampling

**FIGURE 1:**
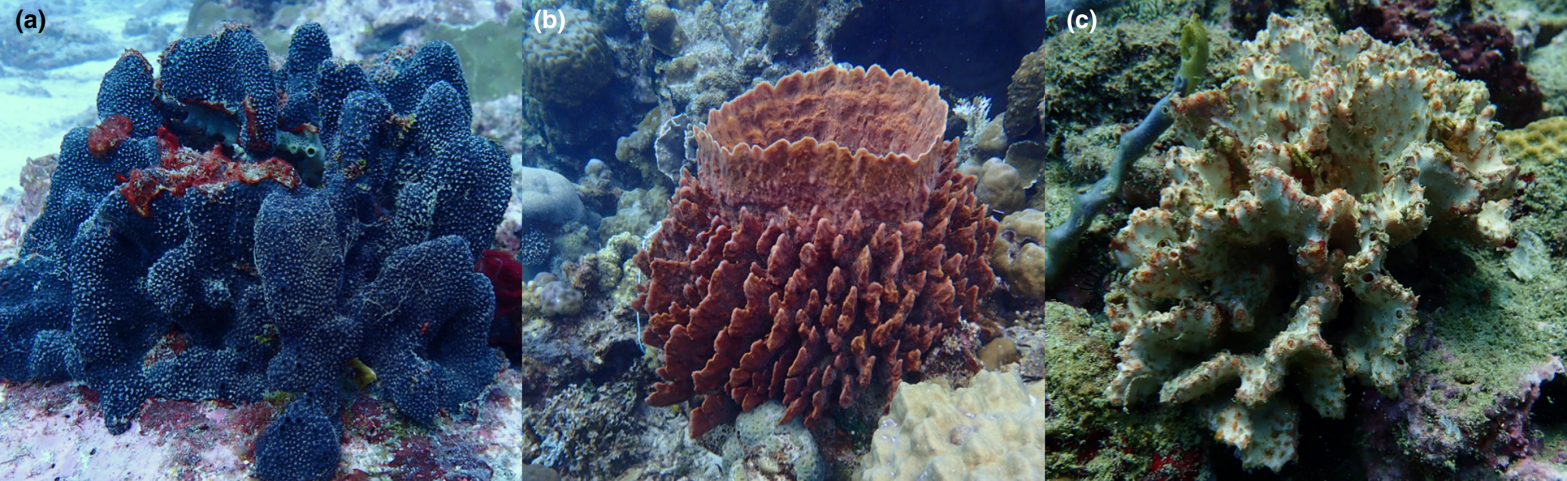
Images of (a) *Hyrtios erectus*, (b) *Xestospongia testudinaria* and (c) *Stylissa carteri*. Photographs taken by Nicole de Voogd.

**FIGURE 2:**
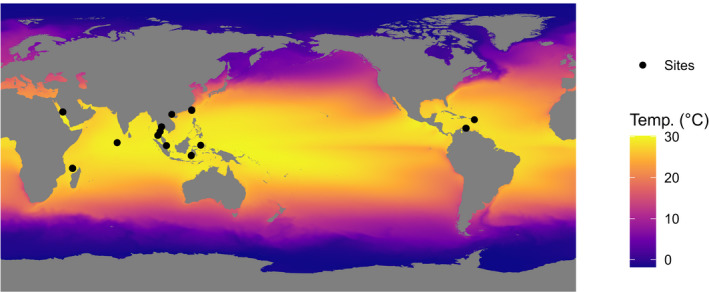
Large scale variation in sea surface temperature (SST); the sampling locations are indicated by black circles. Long‐term mean values of SST for each sample site were obtained from satellite‐derived data, which was downloaded from the National Oceanic and Atmospheric Administration OceanWatch website. This data was imported into R and the extract function from the raster package was used to obtain values for each sample site. Missing values were added using the knnImputation function from the dmwr package with a *K* of 2.

The impact of geographic distance and environmental conditions on the structure of prokaryotic communities was assessed in five biotopes: sediment, seawater and three sponge taxa (Figure 1), namely, the HMA giant barrel sponge (*Xestospongia*) species complex, *X. muta* (Schmidt, 1870) and *X. testudinaria* (Lamarck, 1815), the HMA *Hyrtios erectus* (Keller, 1889) and the LMA *Stylissa carteri* (Dendy, 1889). Samples were collected across the Indo‐Pacific region, and for *X. muta* in the Caribbean, using scuba diving and following previously described protocols (Figure 2) (Cleary et al., 2020; de Voogd et al., 2019). The giant barrel sponges *X. muta* and *X. testudinaria* form a species complex, which includes multiple additional reproductively isolated species (Swierts et al., 2017). A list of the sampling locations is presented in Data S1 including the sampling site and latitude and longitude where each sample was collected. Samples of *Xestospongia* spp. were collected using an apple corer. For the remaining species, samples were collected using a diving knife. Care was taken in order to sample the surface and interior of all sponge specimens. All specimens have been deposited at the sponge collection of Naturalis Biodiversity Center.

### 2.2 DNA extraction

Total community DNA was isolated from all samples using the FastDNA SPIN soil kit following the manufacturer’s instructions. Sediment samples were prepared by centrifuging each sample for 30 min at 4400 rpm and 4°C. For seawater and sponge samples, the membrane filters (seawater) or sponge specimens were each cut into small pieces. Care was taken to ensure that the sponge samples included outer and inner tissues. The whole membrane filter and ±500 mg of sediment or sponge were transferred to Lysing Matrix E tubes containing a mixture of ceramic and silica particles. The microbial cell lysis was performed in the FastPrep Instrument (Q Biogene) for 80 s at 6.0 m/s. The extracted DNA was eluted into DNase/Pyrogen‐Free Water to a final volume of 50 μl and stored at −20°C until use. The 16S rRNA gene V3V4 variable region PCR primers 341F 5′‐CCTACGGGNGGCWGCAG‐3′ and 785R 5′‐GACTACHVGGGTATCTAATCC‐3′ (Klindworth et al., 2013) with barcode on the forward primer were used in a 30 cycle PCR assay using the HotStarTaq Plus Master Mix Kit (Qiagen) under the following conditions: 94°C for 3 min, followed by 28 cycles of 94°C for 30 s, 53°C for 40 s and 72°C for 1 min, after which a final elongation step at 72°C for 5 min was performed. After amplification, PCR products were checked on a 2% agarose gel to determine the success of amplification and the relative intensity of bands. PCR products were used to prepare the DNA library following the Illumina TruSeq DNA library preparation protocol. Next‐generation, paired‐end sequencing was performed on an Illumina MiSeq device using MiSeq reagent kit version 3 (Illumina Inc.) at MrDNA (http://www.mrdna.org/; last checked 19 December 2020). Sequences from each end were joined following Q25 quality trimming of the ends followed by reorienting any 3′–5′ reads back into 5′–3′ and removal of short reads (<150 bp).

### 2.3 Sequencing analysis

The 16S rRNA amplicon libraries were analysed using qiime2 (version 2019.7; Bolyen et al., 2019). Raw data was imported yielding a demultiplexed “qza” data file (artefact). The DADA2 plugin (Callahan et al., 2016) in qiime2 was subsequently used to trim sequences (final length 400 nt). The DADA2 analysis yielded output archives containing an operational taxonomic unit (OTU) (also known as amplicon sequence variant or ASV) table, denoising stats and a fasta file of representative sequences. The feature‐classifier plugin with the extract‐reads method was then used with the i‐sequences argument set to silva‐138‐99‐seqs.qza. This was followed by the feature‐classifier plugin with the fit‐classifier‐naive‐bayes method and the i‐reference‐taxonomy method set to silva‐138‐99‐tax.qza. Both silva‐138 files can be obtained from https://docs.qiime2.org/2020.8/data‐resources/?highlight=silva. The feature‐classifier plugin was then used with the classify‐sklearn method and the i‐reads argument set to the representative sequences file generated by the DADA2 analysis to produce a table with taxonomic assignments for all OTUs. Finally, mitochondria, chloroplasts, and Eukaryota were filtered out using the qiime taxa plugin with the filter‐table method. The OTU and taxonomy tables were later merged in R (R Core Team, 2022). After denoising, removal of chimera, mitochondria, chloroplasts, and Eukaryota, the final data set consisted of 1,926,144 sequences and 18,940 OTUs.

### 2.4 Remotely sensed data

Global remotely sensed data was downloaded from the National Oceanic and Atmospheric Administration OceanWatch website (https://oceanwatch.pifsc.noaa.gov/erddap/index.html; last checked 2020 12 11). This consisted of cumulative long term mean (January 2003–February 2019) Aqua MODIS data for Chlα and Coral Reef Watch data (2003–2017) for SST. All data was downloaded in netCDF format and imported into R using the raster function from the raster package. Missing values were added using the knnImputation function from the dmwr package (Torgo, 2016) with a *K* of 2. A Map of the study area showing long‐term variation in SST including the locations of the sampling sites is shown in Figure 1. Data S2 contains R code to download and analyse the remotely sensed data sets.

### 2.5 Genetic differences between pairs of *Xestospongia* spp. samples

DNA was extracted from sponge tissue using the DNeasy Blood and Tissue kit (Qiagen) following the manufacturer’s instructions. For the cytochrome *c* oxidase subunit 1 (CO1) gene, the primers C1‐J2165 (5′‐GAAGTTTATATTTTAATTTTACCDGG‐3′) and C1‐Npor2760 (5′‐TCTAGGTAATCCAGCTAAACC‐3′) were used, which amplified a fragment of 544 base pairs (bp). For further details see Swierts et al. (2013, 2017). The sequences (Data S3) obtained were used to generate a genetic distance/difference matrix with the dist.hamming function in the R‐package phangorn (Schliep, 2011). The hamming function is based on the Hamming distance, which is a metric describing the minimum number of mutations required to convert one sequence into another sequence (Hamming, 1950).

### 2.6 Statistical analysis

Different analytical techniques can be employed to study beta diversity. Constrained ordination techniques, such as canonical correspondence analysis or redundancy analysis (RDA), for example, can be used to relate communities of organisms to spatial and/or environmental gradients. The variance explained in such ordination techniques is proportional to the variance in species abundances explained by the environmental or spatial data. In contrast, multiple regression on distance matrices (MRM; Lichstein, 2007) quantifies the amount of variance in pairwise dissimilarity values, which can be explained by spatial and/or environmental data and as such addresses differences in composition at different sites, as opposed to differences in the abundance of species among sites (Tuomisto & Ruokolainen, 2006). Both of these methods can be considered complementary approaches, which help to understand the origin of beta diversity. In addition to the above, zeta diversity (Hui & McGeoch, 2014) is a more recent addition to the means of assessing beta diversity and addresses some of the limitations of pairwise metrics.

Tables containing the OTU counts and geographic coordinates were imported into R using the read.csv function. Data S4 contains all OTU counts per sample and taxonomic assignments of all OTUs. Data S5 contains all 16S partial sequences of the aforementioned OTUs. A Bray‐Curtis distance matrix was obtained using the phyloseq package whereby the count data was first rarefied using the rarefy_even_depth function with the sample.size argument set to the minimum sample size (16,896 in the present study) and subsequently log_10_ transformed. For geographic distance, a distance matrix was obtained using the earth.dist function from the fossil package. This creates a distance matrix of pairwise distances in kilometres between all sample points. The environmental data matrices consisted of distance matrices obtained using the vegdist function in vegan with the method argument set to “euclidean”. Relationships between disssimilarity and geographic distance have been shown to follow power laws for a wide range of taxa (Clark et al., 2021). In the present study, we applied the Levenberg–Marquardt algorithm using the nlsLM function from the minpack.lm library in R to estimate the parameters of power law models of dissimilarity versus the geographic distance between sampling sites for each biotope. For each biotope separately, the MRM function from the ecodist library in R was, furthermore, used with 1000 permutations to carry out multiple regressions on distance matrices with the Bray–Curtis dissimilarity matrices as response variables and spatial (geographic distance) and environmental (SST and Chlα) data matrices as predictor variables. The Bray–Curtis dissimilarity and geographic distance matrices were log_10_ transformed. A matrix of genetic distances/differences was included as an additional predictor for *Xestospongia* spp. Finally, the diffslope function in the simba package in R was used to test for significant differences in the slopes of dissimilarity versus geographic distance between sampling sites for pairs of biotopes.

In addition to MRM, constrained ordination with RDA was used to assess to what extent geographic distance and environmental variables (SST and Chlα) were able to predict variation in the OTU composition of all five biotopes. Spatial variation across the study area was modelled using principal coordinates of neighbour matrices (PCNM). PCNM is a method for quantifying spatial trends across a range of scales and is based on eigenvalue decomposition of a truncated matrix of geographic distances among sampling sites (Borcard & Legendre, 2002). For a detailed description of PCNM, see Borcard and Legendre (2002). PCNM eigenvectors were selected using the pcnm function from the vegan package in R. The OTU table was log_10_ (*x* + 1) transformed and further “transformed” using the decostand function in vegan. With the decostand transformation, the species data were adjusted so that subsequent ordination analyses preserved the chosen distance among sample sites. In the present case, the Hellinger distance was used, as recommended by Legendre and Gallagher (2001). For each biotope, models were set up using RDA with the Hellinger‐transformed matrix as response variable and PCNM, SST and Chlα as explanatory variables. Prior to model selection, significant PCNM vectors were selected using the forward.sel function from the adespatial package (Dray et al., 2020). RDA arranges data points in a multidimensional space where the axes represent gradients in species abundances, constrained by the explanatory variables (PCNM, SST and Chlα) (Makarenkov & Legendre, 2002). The amount of variation in composition explained by the explanatory variables is the sum of all constrained eigenvalues divided by the total variation in the OTU data. Finally, we computed regression models of zeta diversity using the Zeta.msgdm function in the zetadiv library in R. In the Zeta.msgdm function, the reg.type argument was set to ispline, the rescale argument to TRUE, the order argument to 3, the distance type argument to ortho and the normalize argument to Sorensen. Separating the contribution of space and the contribution of environment to patterns of community dissimilarity is necessary for understanding the mechanisms that structure communities across land and seascapes (Spencer et al., 2002). Variance partitioning (Borcard & Legendre, 2002; Borcard et al., 1992; Cleary et al., 2004, 2005) was used to partition the variance explained (1) purely by geographic distance (or PCNM), (2) purely by environmental variables and (3) by combinations of geographic distance and environmental variables. The variance partitioning for *Xestospongia* spp. also included a genetic difference component represented by a matrix of genetic distances between pairs of sponge individuals.

## 3 RESULTS

**FIGURE 3:**
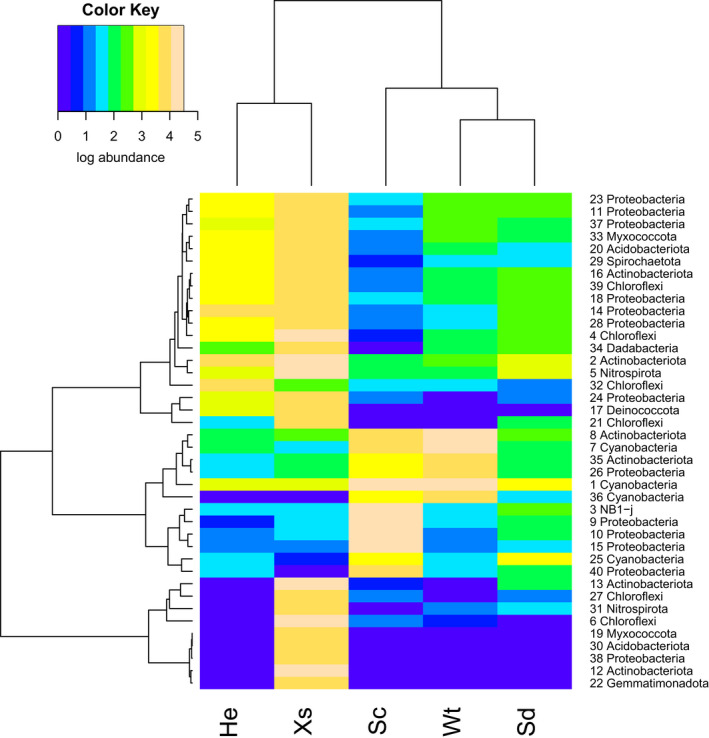
Heatmap of the most abundant OTUs found in Xs: *Xestospongia* spp., He: *Hyrtios erectus*, Sc: *Stylissa carteri*, Sd: Sediment and Wt: seawater. The colour key at the top left of the figure represents abundance on a log_10_ scale. The dendrograms for rows and columns were generated using the hclust function in R, which applies a hierarchical cluster analysis based on a Bray–Curtis dissimilarity matrix of the most abundant OTUs and Ward's clustering function. The row labels refer to OTUs and their assigned phyla.

**FIGURE 4:**
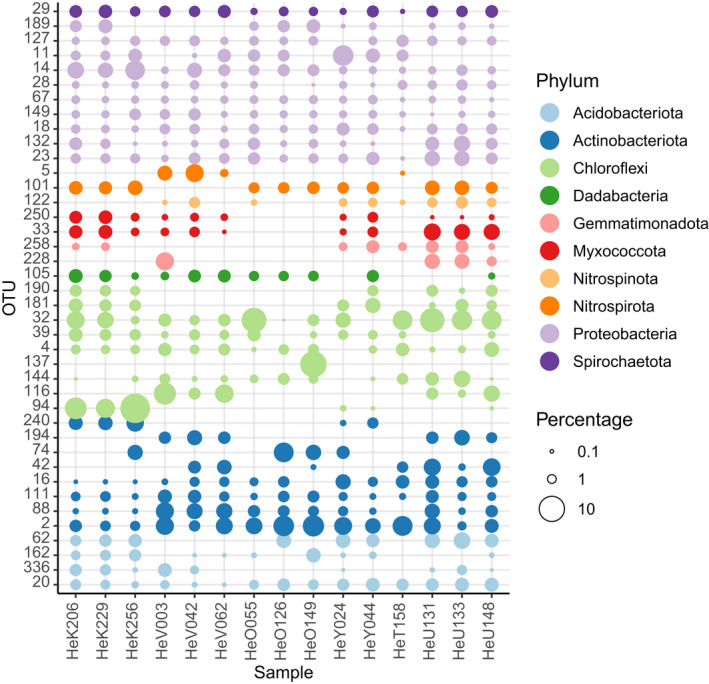
Symbols represent OTUs (along the *y*‐axis) found in samples of *H. erectus* and colour‐coded according to prokaryotic phylum assignment. The circle size of the OTU is proportional to the mean percentage of sequences per sample as indicated by the symbol legend at the right of the figure.

**FIGURE 5:**
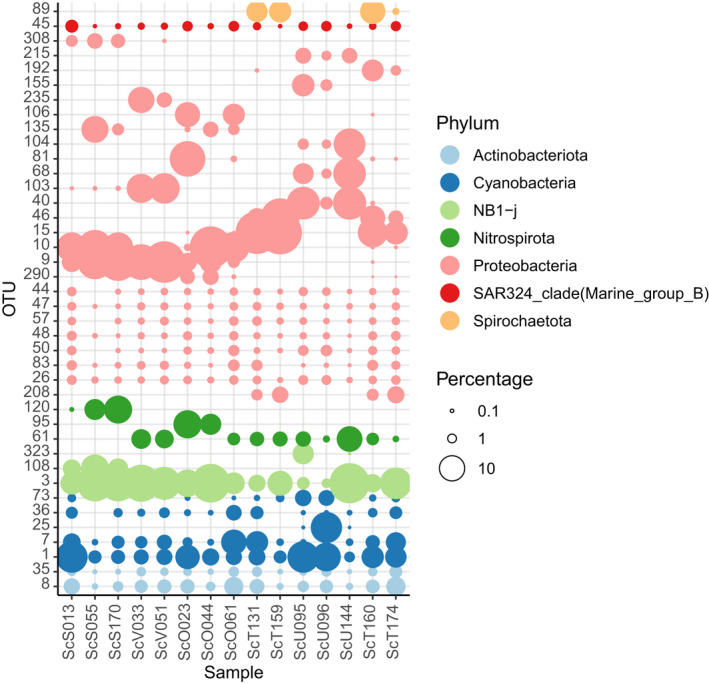
Symbols represent OTUs (along the *y*‐axis) found in samples of *S. carteri* and colour‐coded according to prokaryotic phylum assignment. The circle size of the OTU is proportional to the mean percentage of sequences per sample as indicated by the symbol legend at the right of the figure.

A heatmap of the distribution of the 40 most abundant OTUs revealed that most of the abundant OTUs were recorded in multiple biotopes, but with pronounced variation in abundance across biotopes (Figure 3). Figures 4 and 5, which show the most abundant OTUs of *H. erectus* and *S. carteri*, respectively, highlight the pronounced differences in the structure of the prokaryotic communities of both species.

**FIGURE 6:**
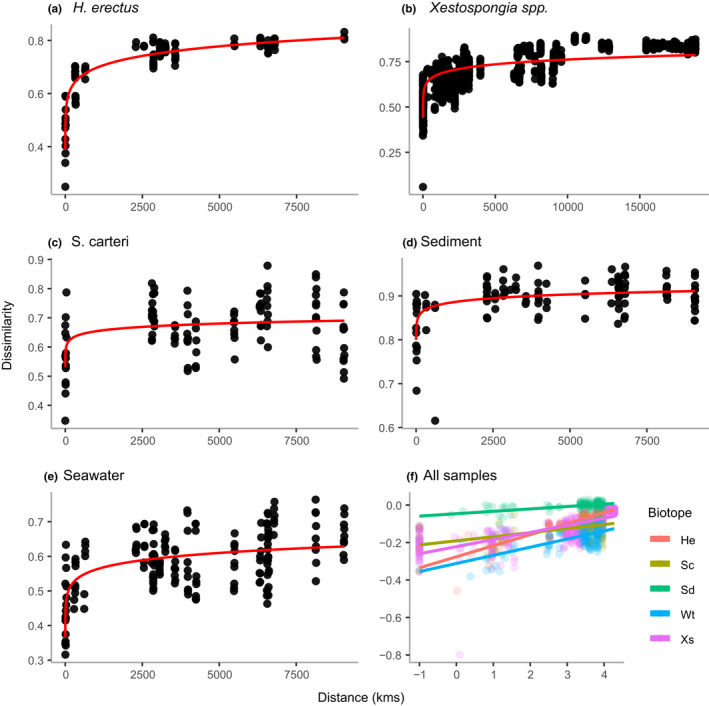
Variation in community dissimilarity as a function of distance between sampling sites for: (a) *Hyrtios erectus*, (b) *Xestospongia* spp., (c) *Stylissa carteri*, (d) sediment, (e) seawater and (f) all biotopes plotted using a log–log scale. Each point represents a single pairwise comparison of Bray–Curtis dissimilarity between pairs of sites. The fitted lines were obtained using power law functions for (a–e) and linear regression for each biotope in (f).

Spatial and environmental variables explained significant amounts of variation in dissimilarity in all biotopes. The amount of variation explained varied from ~20% for *S. carteri* (MRM: *F* = 13.05*, p* < .001, *R* ^2^ = .204), ~27% for sediment (MRM: *F* = 21.18, *p* < .001, *R* ^2^ = .266), ~43% for water (MRM: *F* = 64.20*, p* < .001, *R* ^2^ = .433), ~58% for *Xestospongia* spp. (MRM: *F* = 537.88*, p* < .001, *R* ^2^ = .579, including genetic difference in addition to spatial and environmental variables) to ~82% for *H. erectus* (MRM: *F* = 227.01*, p* < .001, *R* ^2^ = .817). In Figure 6a–e, power law curves are fit to graphs of dissimilarity versus the geographic distance between sampling sites for all five biotopes. In Figure 6f samples from all biotopes are plotted together on a log–log scale. In Figure 6f, the slope of *H. erectus* (0.059) was significantly greater than all other biotopes (diffslope: *all p* < .05). The slope of seawater (0.043) was significantly greater than sediment (diffslope*: p* < .001) and *S. carteri* (diffslope*: p* = .015), but did not differ significantly from that of *Xestospongia* spp. (diffslope*: p* = .082). The slope of *Xestospongia* spp. (0.038) was significantly greater than sediment (diffslope*: p* < .001) and *S. carteri* (diffslope: *p* < .001). There was no significant difference (diffslope: *p* = .163) between the slopes of sediment (0.013) and *S. carteri* (0.22).

**FIGURE 7:**
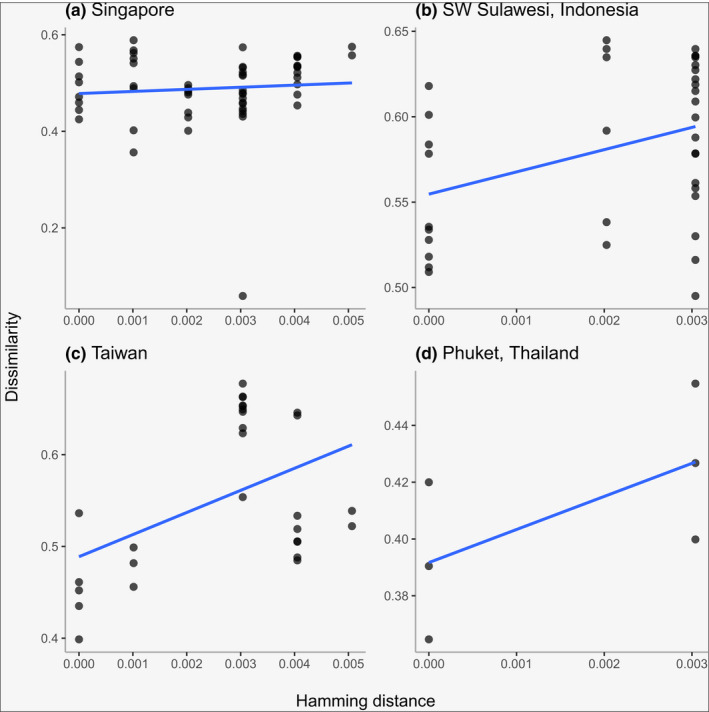
Variation in community dissimilarity as a function of genetic difference between pairs of Xestospongia spp. samples. Results are shown for samples collected in (a) Singapore, (b) Spermonde Archipelago, SW Sulawesi, Indonesia, (c) Taiwan and (d) Phuket, Thailand. The fitted lines were obtained with linear regression in R.

As noted above, genetic difference was included as a predictor of variation in prokaryotic composition for the *Xestospongia* species complex. Across all samples, genetic difference explained >7% of variation in dissimilarity (MRM: *F* = 96.30*, p* < .001, *R* ^2^ = .076). At local scales, the univariate relationships between dissimilarity and genetic difference were non‐significant for samples from Singapore (*F* = 0.06*, p* = .847, *R* ^2^ = .001) and Phuket (*F* = 2.48*, p* = .273, *R* ^2^ = .383), but significant for samples from the Spermonde archipelago, Indonesia (*F* = 5.36*, p* = .023, *R* ^2^ = .136) and Taiwan (*F* = 8.26*, p* = .018, *R* ^2^ = .241) (Figure 7).

**FIGURE 8:**
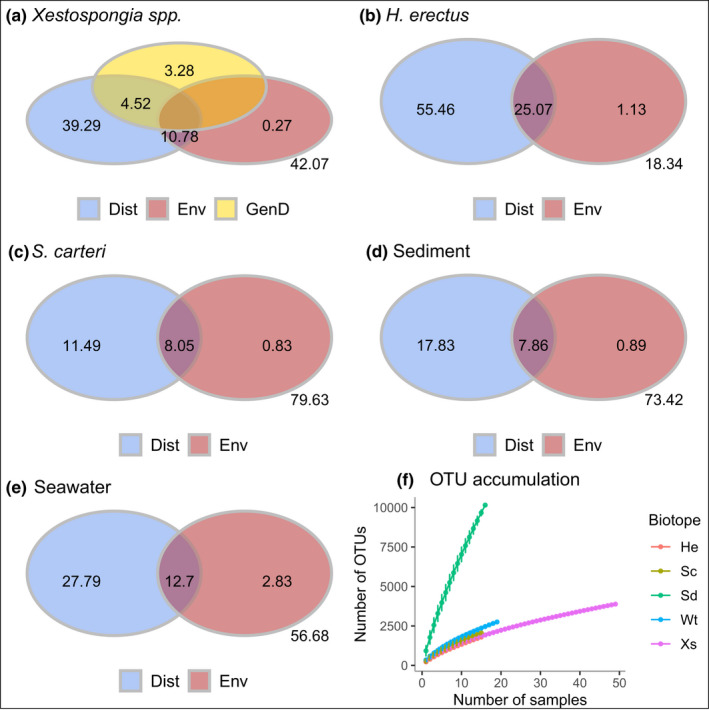
Venn diagrams showing the results of variance partitioning based on MRM analyses for (a) *Xestospongia* spp., (b) *Hyrtios erectus*, (c) *Stylissa carteri*, (d) sediment and (e) seawater. Cumulative richness curves are shown for all biotopes in (f); the bars for each sample represent the standard error of the estimate. In figure (a–e) overlapping areas indicate the percentage of variation in dissimilarity explained by the union of different factors. Nonoverlapping areas indicate purely spatial (Dist), purely environmental (Chlα and SST) or purely genetic (in the case of *Xestospongia* spp.) components.

Results of the variance partitioning analyses are shown in Figure 8. Taken together, geographic distance, SST, Chlα, and genetic difference (for *Xestospongia* spp.) explained from just over 20% (*S. carteri*) to >80% (*H. erectus*) of the variation in dissimilarity. The purely spatial component, after partitioning out the variation explained by environmental variables and genetic difference, explained from 11.49% (*S. carteri*) to 55.46% (*H. erectus*) of variation in dissimilarity. The purely environmental component explained from 0.15% (*H. erectus*) to 2.83% (seawater) of variation in dissimilarity while the union of geographic distance and environment explained from 7.86% (sediment) to 25.07% (*H. erectus*). For *Xestospongia* spp., the purely spatial component explained 39.29%, the purely environmental component 0.27%, the purely genetic component 3.28%, the union of geographic distance and genetic difference 4.52%, and the union of geographic distance and environment 10.78% of the variation in prokaryotic dissimilarity.

With constrained ordination, the full set of spatial (PCNM vectors) and environmental variables proved significant predictors of variation in prokaryotic composition for all five biotopes (RDA: *Xestospongia* spp.: *F* = 3.345*, p* < .001; *H. erectus*: *F* = 2.959*, p* < .001; *S. carteri*: *F* = 1.842*, p* < .001; seawater: *F* = 2.28*, p* < .001 and sediment: *F* = 1.284*, p* < .001). The full set of predictors (distance and environment) explained 24.51% of the variation in prokaryotic composition of sediment, 40.09% of *Xestospongia* spp., 42.42% of *S. carteri*, 53.28% of seawater and 68.93% of *H. erectus*. The purely spatial component (PCNM variables after partialling out the variation explained by SST and Chlα) explained 9.39% of the variation in composition of sediment, 20.25% of *S. carteri*, 27.35% of *Xestospongia* spp., 35.57% of seawater and 45.46% of *H. erectus*. Values for the other components are shown in Table 1.

**TABLE 1:** Results of variance partitioning analyses comparing MRM, constrained ordination and zeta diversity approaches.

Biotope	Approach	Purely spatial	Spatial and environmental	Purely environmental	Explained total	Unexplained
*Xestospongia* spp.	MRM	43.81	10.68	0.15	54.64	45.36
	Constrained	27.35	5.39	7.35	40.09	59.91
	Zeta	14.37	14.05	26.12	54.54	45.46
*H. erectus*	MRM	55.46	25.07	1.13	81.66	18.34
	Constrained	45.46	4.64	18.83	68.93	31.07
	Zeta	19.55	20.03	29.69	69.27	30.73
*S. carteri*	MRM	11.49	8.05	0.83	20.37	79.63
	Constrained	20.25	6.67	15.5	42.42	57.58
	Zeta	4.45	5.92	14.31	24.68	75.32
Seawater	MRM	27.79	12.7	2.83	43.32	56.68
	Constrained	35.57	2.13	15.58	53.28	46.72
	Zeta	7.17	3.37	9.96	20.5	79.5
Sediment	MRM	17.83	7.86	0.89	26.58	73.42
	Constrained	9.39	0	15.12	24.51	75.49
	Zeta	1.47	0.93	7.94	10.34	89.66

In the zeta diversity analysis, spatial and environmental variables (Table 1) explained 10.34% of the variation in prokaryotic composition of sediment, 20.5% of seawater, 24.68% of *S. carteri*, 55.54% of *Xestospongia* spp. and 69.27% of *H. erectus*. The purely spatial component explained 1.47% of the variation in composition of sediment, 4.45% of *S. carteri*, 7.17% of seawater, 14.37% of *Xestospongia* spp. and 19.55% of *H. erectus*.

All three approaches (MRM, constrained ordination and zeta diversity) aligned in the purely spatial component (geographic distance alone) being more important for the HMA taxa *Xestospongia* spp. and *H. erectus* than for the LMA *S. carteri*. The zeta diversity approach also had stronger spatially‐structured and purely environmental components for both HMA species compared to the other biotopes. Finally, cumulative richness differed markedly among biotopes with rarefied richness for 15 samples: 1849.85 ± 122.94 OTUs for *Xestospongia* spp., 1792 ± 0 for *H. erectus*, 2063 ± 0 for *S. carteri*, 2362.98 ± 142.31 for seawater and 9667.99 ± 216.68 for sediment (Figure 8).

## 4 DISCUSSION

Recent studies have shown there to be considerable variation in distance‐dependence across different taxonomic groups, scales and ecosystems (Clark et al., 2021). In a comparison of the impact of environmental variables and geographic distance on variation in dissimilarity, Hanson et al. (2012) found that, on average, environmental variables explained 26.9% of the variation in dissimilarity compared to 10.3% explained by geographic distance for microbial communities. Together, environment and distance (distance and environment alone in addition to the union of both) explained on average 49.7% of the variation in dissimilarity. Hanson et al. (2012) noted that this was remarkably similar to the amount of variation explained for communities of multicellular organisms where an average of 22% was explained by environment alone, 16% by geographic distance alone and 48% by spatial and environmental factors combined.

Although, numerous studies to date have observed distance‐dependence in microbial communities (Hanson et al., 2012 and references therein; Chen et al., 2020; Clark et al., 2021; Díez‐Vives et al., 2020; Hou et al., 2020; Locey et al., 2020; Wang et al., 2020), theory predicts that it should be relatively weak in highly connected, marine environments as opposed, for example, to grassland‐based studies (Hanson et al., 2012). In contrast to these expectations, however, Clark et al. (2021) found stronger distance‐dependence in aquatic (marine and freshwater) environments than in terrestrial (grassland) systems. Likewise, Clark et al. (2021) found that distance‐dependence differed significantly between host‐associated and water‐based communities in marine environments with distance‐dependence stronger, albeit highly variable, in host‐associated communities.

In the present study, distance‐dependence was stronger for both HMA taxa and seawater than for the LMA *S. carteri* and sediment. The HMA taxa included the multi‐species *Xestospongia* species complex, which consisted of samples of *X. testudinaria* and *X. muta* sampled in the Indo‐Pacfic and Caribbean. Both of these taxa are believed to have been separated for at least 18–12 Ma since the closing of the Tethys Seaway (Adams et al., 1983; Swierts et al., 2017). Swierts et al. (2017), furthermore, provided evidence for the existence of multiple additional species in both the Indo‐Pacific and Caribbean. We, thus, included genetic difference as a predictor of variation in the composition of the *Xestospongia* species complex. Various studies have shown that host genetics play an important role in structuring the microbiomes of a number of organisms (Díez‐Vives et al., 2020 and citations therein). Although host identity proved to be the most important determinant of microbial composition, Thomas et al. (2016) showed that certain phylogenetically‐related species also housed similar microbial communities. In this study, although significant, the genetic difference between sponge individuals only explained a relatively small portion (<8%) of the variation in dissimilarity of the prokaryotic communities of the *Xestospongia* species complex. Only ~3% of the variation in dissimilarity was, furthermore, attributed to genetic difference alone after partialling out the variation due to geographic distance.

In order to assess to what extent the impact of genetic difference on prokaryotic composition may be scale dependent, we assessed four local subsets of sites, namely, Singapore, the Spermonde Archipelago (Makassar, SW Sulawesi, Indonesia), Phuket (Thailand) and Taiwan. The Taiwan data set was particularly interesting because all specimens were collected at a single site within tens of meters of the other specimens. This sampling strategy should greatly reduce the importance of geographic distance. Indeed, the amount of variation explained by genetic difference was relatively high (~24%) in Taiwan. This suggests that prokaryotic community composition may be structured by different processes at small and large spatial scales with differences attributable to host genotype more important at very small spatial scales. However, in two of the areas (Singapore and Phuket), there were no significant associations between prokaryotic dissimilarity and genetic difference. This was probably, at least partly, attributable to under‐sampling at these locations, particularly for Phuket where the amount of variation explained by genetic difference was high (~38%). The population of *Xestospongia* sponges in Singapore, furthermore, may be subject to pronounced disturbance given their proximity to the city of Singapore and associated chronic perturbations (Tan et al., 2016). Disturbance may, thus, help to explain the lack of a relationship between genetic difference and prokaryotic dissimilarity although this is speculative. Future research should increase sampling effort at smaller spatial scales and in different environments in order to robustly test if the impact of genetic difference on prokaryotic composition is indeed scale dependent.

In line with the rapid rise in dissimilarity at the smallest spatial scales observed in our study, previous studies of plant and animal communities also observed rapid changes in dissimilarity (or similarity) at very small spatial scales. For example, Cleary et al. (2004) showed rapid distance‐decay in similarity for butterflies and odonates at distances <10 km. Tuomisto et al. (2003) noted that geographic distance had the clearest effect at distances <80 km with a rapid decline in floristic similarity despite mean environmental similarity remaining relatively unchanged.

A number of studies have, furthermore, shown that taxon accumulation with increasing area and distance decay relationships are lower for microbial communities than for communities of plants and animals (Green et al., 2004; Hillebrand et al., 2001; Horner‐Devine et al., 2004; Meyer et al., 2018). This has been attributed to fundamental differences between microbes on the one hand and multicellular organisms on the other. There has, however, been considerable debate about the degree to which the geographic patterns of microorganisms differ from those of plants and animals (Astorga et al., 2012). This is related to the ongoing “Everything is everywhere, but the environment selects” (Baas Becking, 1934) debate where the population sizes and dispersal abilities of microorganisms are believed to limit or eliminate the role of purely spatial processes in structuring microbial communities (Godfray & Lawton, 2001). Differences between both groups, however, may be an artefact of how microbial communities are generally studied including the suggestion that OTUs, or ASVs, may not represent an appropriate analogue of plant and animal species (Meyer et al., 2018). Dormancy is also an important attribute of microbial communities, which is much less prevalent in animal communities, although the seed banks of plants are equivalent.

Various microbes have the ability to enter dormancy, a state of reduced metabolic activity, which enables them to weather adverse environmental conditions. Together these species form a microbial seed bank limiting the impact of environmental filtering and increasing potential dispersal (Locey et al., 2020). Dormancy may be expected to be particularly prevalent in the sediment community. By reducing the effect of environmental filtering and dispersal limitation, dormancy should reduce distance dependence and thus the slope of distance‐dissimilarity plots (Locey et al., 2020). In contrast to this expectation, Meyer et al. (2018) found that the active soil bacterial community had a significantly flatter slope than the total community. Meyer et al. (2018), furthermore, noted that neither a broader similarity cutoff (e.g., 95%) nor a narrower cutoff (99 to 100%) significantly affected distance‐dependence.

In contrast to dormancy, Meyer et al. (2018) found that undersampling did significantly affect distance‐dependence. The effect of undersampling (or incomplete sampling) was most pronounced for highly diverse microbial communities. Meyer et al. (2018) attributed this to positive frequency abundance relationships of taxa in the community whereby lesser abundant, range‐restricted taxa will be missed from surveys thus leading to an underestimate of turnover. Most studies of microbial communities have species accumulation curves, which are far from saturation (Meyer et al., 2018; Rosenzweig, 1995; Woodcock et al., 2006). In the present study, this was most pronounced for the sediment prokaryotic community, which accumulated much more OTUs than the *Xestospongia* species complex. Our results, furthermore, confirmed previous studies with the sediment community having a much higher diversity than sponge‐associated prokaryotic communities (Cleary et al., 2019, 2020; Polónia et al., 2014, 2017; Polónia, Cleary, Freitas, et al., 2015).

Neutral theory predicts the distance‐dependence of dissimilarity due to historical processes and independence of environmental control (Hubbell, 2001). Astorga et al. (2012; and citations therein), however, noted that neutral dynamics and environmental control represent opposite ends of a continuum with most natural communities somewhere between these two extremes. Environmental variables also often exhibit distance‐dependence rendering it difficult to disentangle the exact mechanisms structuring communities (Gilbert & Lechowicz, 2004). Neutral theory also predicts that distance‐dependence should be strongest at the smallest spatial scales (Hubbell, 2001; Jones et al., 2006). Hubbell (2001) suggested that the steep decline in similarity (or increase in dissimilarity) at relatively short distances was caused by rare species. Morlon et al. (2008), however, showed that removing all species with fewer than 50 individuals had relatively little effect on distance‐dependence while removing just 2% of abundant species had a substantial effect. In the present study, geographic distance proved a significant predictor of variation in dissimilarity for all biotopes. Environmental variables explained a significant, albeit, lower amount of variation using MRM and constrained ordination, but not zeta diversity where the purely environmental component was greater than the purely spatial component. Substantial variation remained unexplained in all biotopes. This unexplained variation may be due to stochastic processes, historical factors such as major disturbances and unmeasured but important environmental factors. Potentially important environmental variables for sponges and their microbiomes include salinity, pH, nutrient concentrations, the concentration and composition of dissolved organic matter (e.g., humic substances), substrate composition and water transparency among others. It is, however, important to note that the importance of these variables may vary with scale, with certain variables more important at very small spatial scales and others at larger spatial scales.

Condit et al. (2002) suggested that the rapid decline in similarity (or increase in dissimilarity) at relatively short distances was due to species being more aggregated at these distances than predicted by theory. Morlon et al. (2008), likewise, suggested that steep distance‐decay relationships reflect aggregation of abundant species as opposed to spatial turnover and that overall dissimilarity is a better descriptor of beta diversity. Morlon et al. (2008) suggested that studies of distance‐dependence should expand their focus to include intercepts, half‐distances and average dissimilarities. The greater height of the curve indicates that composition is more distinct and thus variable in the prokaryotic communities of sediment. This is in line with the greater cumulative richness of sediment than the remaining biotopes. The sediment community, and that of *S. carteri*, also had the shallowest slopes of all communities.

The slope of the distance‐dissimilarity relationship itself is, thus, not necessarily a good indicator of species turnover and gamma diversity, that is, total species richness (Morlon et al., 2008). For example, it was previously believed that shallow distance‐decay slopes were reflective of low species turnover and thus relatively low richness at large spatial scales (Harte et al., 1999; Morlon et al., 2008). However, in this study, the sediment community was by far the richest community, but had the shallowest slope. In contrast, *H. erectus* had the steepest slope but the lowest number of accumulated OTUs. In line with this, Woodcock et al. (2006) showed that taxa can accumulate with area even under a flat distance decay relationship.

The importance of geographic distance in explaining variation in the dissimilarity of *H. erectus* may not be (solely) due to dispersal limitation of the OTUs in question, but rather reflect spatial processes related to mass and founder effects (priority effects) (Guélat et al., 2008; Leibold et al., 2004). The limited richness across very large spatial scales may reflect filtering by the host sponge (Hadas et al., 2006; Kowalke, 2000; Pile et al., 1996; Reiswig, 1971, 1975, 1990; Ribes et al., 1999) or habitat specificity where only subsets of potential colonizers are able to successfully colonize the distinct, HMA sponge environment. This would represent a form of constrained stochasticity whereby there are a limited number of potential colonizers, but composition is largely structured by spatial processes. This may also include priority effects, emphasizing the importance of initial colonizers (Maas et al., 2018). Initial colonization may yield sufficient numerical dominance to limit subsequent colonizing attempts by other species/taxa. This may be followed by rapid adaptation to the host organism, further strengthening competitive exclusion, which may in turn be countered by mass effects. Significant distance‐dependence may, thus, not (only) be due to dispersal limitation, but also related to priority effects, drift and even previous selection, collectively known as historical processes (Hanson et al., 2012).

Previous studies have shown pronounced similarity of the prokaryotic communities of phylogenetically distinct, HMA sponges (Bayer et al., 2014; de Voogd et al., 2019; Moitinho‐Silva et al., 2017). HMA sponges have also been shown to share certain physiological and morphological traits, such as relatively small choanocyte chambers and a massive or tubular growth form with a high mesohyl density; they can also be abundant components of marine ecosystems (Vacelet & Donadey, 1977; Weisz et al., 2008; Wilkinson, 1978). Cleary et al. (2019) showed that the prokaryotic communities of HMA species are, furthermore, highly distinct, both from LMA sponge species as well as from other host organisms including hard and soft corals, sea cucumbers and sea urchins in addition to sediment and seawater.

With respect to our initial hypotheses, it would appear that hypothesis 2, namely that composition is spatially autocorrelated applies to all biotopes, but most strongly to the HMA sponges and seawater. The possible reasons for this have been discussed above. For *S. carteri*, in contrast, a combination of hypotheses 1 and 2 appears to be more appropriate. Hypothesis 1 emphasizes uniform composition and thus biotic interactions and dominance of a limited set of competitively dominant species. This is reflected in the very different distributions of abundant OTUs between *H. erectus* and *S. carteri*. The small subset of dominant OTUs in *S. carteri* were also rare components of other biotopes. *S. carteri* also shared a number of abundant OTUs with seawater. Certain taxa, which were relatively abundant in seawater, were also well represented in *S. carteri* (e.g., Cyanobacteria) whereas others were underrepresented (e.g., Bacteroidetes and Euryarchaeota). It is not yet known whether these taxa play an important functional role in the sponge (although the abundance of Cyanobacteria suggests there is) or are simply seawater microbes, which were selectively filtered by *S. carteri* for consumption. However, in a study of the functional gene repertoire of *S. carteri* from the Red sea, it was shown that most of the abundant gene functions assigned to Cyanobacteria in *S. carteri* were related to photosynthesis and CO_2_ fixation and is thus indicative for the symbiotic role of sponge cyanobacteria (Moitinho‐Silva et al., 2014; Taylor et al., 2007; Wilkinson, 1978).

## 5 CONCLUSION

All biotopes in the present study exhibited distance‐dependence characterized by a relatively rapid rise in dissimilarity at the smallest spatial scales followed by a much more gradual increase in dissimilarity at greater spatial scales. With the MRM and constrained ordination approaches, spatial and environmental variables explained more than 40% of the variation in prokaryotic composition of both HMA sponges and seawater with most of this attributable to the purely spatial component. For *S. carteri*, only 20% of the variation was explained by spatial and environmental variables using the MRM approach with most of this attributable to the purely spatial component. With constrained ordination, in contrast, >40% of variation was explained by spatial and environmental variables with important (>15%) purely spatial and purely environmental components. For sediment, both the MRM and constrained ordination approaches explained relatively low amounts of variation (<27%), but with most of the variation attributable to the purely spatial component with the MRM approach and due to the purely environmental component with the constrained ordination approach. The zeta diversity approach explained considerable amounts of variation in both HMA species (>54%), but less in the other biotopes (<25%). In contrast to the other two techniques, using zeta diversity the purely environmental component explained more variation in all biotopes than the purely spatial component. Based on the above, it is hard to identify the relative importance of spatial versus environmental processes in structuring prokaryotic composition in the host‐associated and abiotic biotopes in the present study. With respect to HMA and LMA species, our results suggest that spatial and environmental factors are more important in structuring the prokaryotic communities of HMA species. This suggests that HMA species may resemble islands whereby the host‐associated prokaryotic communities are more subject to local environmental conditions and spatial processes than LMA species, which are dominated by small subsets of dominant species and other, possibly predominantly transient taxa derived from the pronounced pumping activity associated with LMA species (Cleary et al., 2019, 2020). An important caveat, however, is that we only sampled a single LMA species and had limited environmental variables. Unmeasured environmental variables, for example, may yet prove to be important in structuring the prokaryotic communities of LMA species.

## AUTHOR CONTRIBUTIONS

Daniel F. R. Cleary came up with the idea for the manuscript, and contributed to fieldwork, data analysis, and writing the manuscript. Ana R. M. Polónia contributed to laboratory work, and writing the manuscript. Thomas Swierts contributed to fieldwork, laboratory work and writing the manuscript. Francisco J. R. C. Coelho contributed to writing the manuscript. Nicole J. de Voogd contributed to fieldwork, identification of the sponge specimens and writing the manuscript. Newton C. M. Gomes contributed to writing the manuscript.

## CONFLICT OF INTEREST

The authors declare that they have no conflicts of interest.

## Supporting information


Data S1

Data S2

Data S3

Data S4

Data S5

## Data Availability

The DNA sequences generated in this study can be downloaded from the National Center for Biotechnology Information (NCBI) Sequence Read Archive (SRA) under the codes: PRJNA315454, PRJNA382576, PRJNA397173, PRJNA397175, PRJNA397177, PRJNA397178, PRJNA397180, PRJNA476053.

## References

[mec16631-bib-0001] Adams, C. G. , Gentry, A. W. , & Whybrow, P. J. (1983). Dating the terminal Tethyan event. Utrecht Micropaleontological Bulletins, 30, 273–298.

[mec16631-bib-0002] Astorga, A. , Oksanen, J. , Luoto, M. , Soininen, J. , Virtanen, R. , & Muotka, T. (2012). Distance decay of similarity in freshwater communities: Do macro‐and microorganisms follow the same rules? Global Ecology and Biogeography, 21(3), 365–375.

[mec16631-bib-0003] Baas Becking, L. G. M. (1934). Geobiologie of inleiding tot de milieukunde (No. 18‐19). WP Van Stockum & Zoon.

[mec16631-bib-0004] Bayer, K. , Kamke, J. , & Hentschel, U. (2014). Quantification of bacterial and archaeal symbionts in high and low microbial abundance sponges using real‐time PCR. FEMS Microbiology Ecology, 89(3), 679–690. 24942664 10.1111/1574-6941.12369

[mec16631-bib-0005] Becking, L. E. , Cleary, D. F. R. , de Voogd, N. J. , Renema, W. , de Beer, M. , van Soest, R. W. M. , & Hoeksema, B. W. (2006). Beta diversity of tropical marine assemblages in the Spermonde Archipelago, Indonesia. Marine Ecology – An Evolutionary Perspective, 27, 76–88.

[mec16631-bib-0006] Bolyen, E. , Rideout, J. R. , Dillon, M. R. , Bokulich, N. A. , Abnet, C. C. , al‐Ghalith, G. A. , Alexander, H. , Alm, E. J. , Arumugam, M. , Asnicar, F. , Bai, Y. , Bisanz, J. E. , Bittinger, K. , Brejnrod, A. , Brislawn, C. J. , Brown, C. T. , Callahan, B. J. , Caraballo‐Rodríguez, A. M. , Chase, J. , … Caporaso, J. G. (2019). Reproducible, interactive, scalable and extensible microbiome data science using QIIME 2. Nature Biotechnology, 37, 852–857. 10.1038/s41587-019-0209-9 PMC7015180 31341288

[mec16631-bib-0007] Borcard, D. , & Legendre, P. (2002). All‐scale spatial analysis of ecological data by means of principal coordinates of neighbour matrices. Ecological Modelling, 153(1–2), 51–68.

[mec16631-bib-0008] Borcard, D. , Legendre, P. , & Drapeau, P. (1992). Partialling out the spatial component of ecological variation. Ecology, 73(3), 1045–1055.

[mec16631-bib-0110] Callahan, B. J. , McMurdie, P. J. , Rosen, M. J. , Han, A. W. , Johnson, A. J. A. , & Holmes, S. P. (2016). DADA2: High‐resolution sample inference from Illumina amplicon data. Nature methods, 13(7), 581–583. 27214047 10.1038/nmeth.3869 PMC4927377

[mec16631-bib-0009] Cárdenas, C. A. , González‐Aravena, M. , Font, A. , Hestetun, J. T. , Hajdu, E. , Trefault, N. , Malmberg, M. , & Bongcam‐Rudloff, E. (2018). High similarity in the microbiota of cold‐water sponges of the genus *Mycale* from two different geographical areas. PeerJ, 6, e4935. 29892508 10.7717/peerj.4935 PMC5994334

[mec16631-bib-0010] Chen, L. , Liu, S. , Chen, Q. , Zhu, G. , Wu, X. , Wang, J. , Li, X. , Hou, L. , & Ni, J. (2020). Dispersal limitation drives biogeographical patterns of anammox bacterial communities across the Yangtze River. Applied Microbiology and Biotechnology, 104, 5535–5546. 10.1007/s00253-020-10511-4 32300854

[mec16631-bib-0011] Chow, C. E. T. , Sachdeva, R. , Cram, J. A. , Steele, J. A. , Needham, D. M. , Patel, A. , Parada, A. E. , & Fuhrman, J. A. (2013). Temporal variability and coherence of euphotic zone bacterial communities over a decade in the Southern California Bight. The ISME Journal, 7(12), 2259–2273. 10.1038/ismej.2013.122 23864126 PMC3834854

[mec16631-bib-0012] Clark, D. R. , Underwood, G. J. , McGenity, T. J. , & Dumbrell, A. J. (2021). What drives study‐dependent differences in distance–decay relationships of microbial communities? Global Ecology and Biogeography, 30(4), 811–825. 10.1111/geb.13266

[mec16631-bib-0013] Cleary, D. F. R. , Becking, L. E. , de Voogd, N. J. , Renema, W. , de Beer, M. , van Soest, R. W. , & Hoeksema, B. W. (2005). Variation in the diversity and composition of benthic taxa as a function of distance offshore, depth and exposure in the Spermonde Archipelago, Indonesia. Estuarine, Coastal and Shelf Science, 65(3), 557–570.

[mec16631-bib-0014] Cleary, D. F. R. , de Voogd, N. J. , Polónia, A. R. M. , Freitas, R. , & Gomes, N. C. M. (2015). Composition and predictive functional analysis of bacterial communities in seawater, sediment and sponges in the Spermonde Archipelago, Indonesia. Microbial Ecology, 70(4), 889–903. 10.1007/s00248-015-0632-5 26072397

[mec16631-bib-0015] Cleary, D. F. R. , DeVantier, L. , Giyanto , Vail, L. , Manto, P. , de Voogd, N. J. , Rachello‐Dolmen, P. G. , Tuti, Y. , Budiyanto, A. , Wolstenholme, J. , Hoeksema, B. W. , & Suharsono . (2008). Relating variation in species composition to environmental variables: A multi‐taxon study in an Indonesian coral reef complex. Aquatic Sciences, 70, 419–431.

[mec16631-bib-0016] Cleary, D. F. R. , Mooers, A. Ø. , Eichhorn, K. A. , Van Tol, J. , De Jong, R. , & Menken, S. B. (2004). Diversity and community composition of butterflies and odonates in an ENSO‐induced fire affected habitat mosaic: A case study from East Kalimantan, Indonesia. Oikos, 105(2), 426–448.

[mec16631-bib-0017] Cleary, D. F. R. , Polónia, A. R. M. , Becking, L. E. , de Voogd, N. J. , Gomes, H. , & Gomes, N. C. M. (2018). Compositional analysis of bacterial communities in seawater, sediment, and sponges in the Misool coral reef system, Indonesia. Marine Biodiversity, 48(4), 1889–1901. 10.1007/s12526-017-0697-0

[mec16631-bib-0018] Cleary, D. F. R. , Polónia, A. R. M. , Reijnen, B. T. , Berumen, M. L. , & de Voogd, N. J. (2020). Prokaryote communities inhabiting endemic and newly discovered sponges and octocorals from the Red Sea. Microbial Ecology, 80, 103–119. 10.1007/s00248-019-01465-w 31932882

[mec16631-bib-0019] Cleary, D. F. R. , Polónia, A. R. M. , Renema, W. , Hoeksema, B. W. , Rachello‐Dolmen, P. G. , Moolenbeek, R. G. , Budiyanto, A. , Yahmantoro , Tuti, Y. , Giyanto , Draisma, S. G. , Prud'homme van Reine, W. , Hariyanto, R. , Gittenberger, A. , Rikoh, M. S. , & de Voogd, N. J. (2016). Variation in the composition of corals, fishes, sponges, echinoderms, ascidians, molluscs, foraminifera and macroalgae across a pronounced in‐to‐offshore environmental gradient in the Jakarta Bay–Thousand Islands coral reef complex. Marine Pollution Bulletin, 110(2), 701–717. 10.1016/j.marpolbul.2016.04.042 27179997

[mec16631-bib-0020] Cleary, D. F. R. , Swierts, T. , Coelho, F. J. , Polónia, A. R. M. , Huang, Y. M. , Ferreira, M. R. , Putchakarn, S. , Carvalheiro, L. , van der Ent, E. , Ueng, J. P. , Gomes, N. C. M. , & de Voogd, N. J. (2019). The sponge microbiome within the greater coral reef microbial metacommunity. Nature Communications, 10, 1644. 10.1038/s41467-019-09537-8 PMC6456735 30967538

[mec16631-bib-0021] Condit, R. , Pitman, N. , Leigh, E. G., Jr. , Chave, J. , Terborgh, J. , Foster, R. B. , Núñez, P. , Aguilar, S. , Valencia, R. , Villa, G. , Muller‐Landau, H. C. , Losos, E. , & Hubbell, S. P. (2002). Beta‐diversity in tropical forest trees. Science, 295(5555), 666–669. 11809969 10.1126/science.1066854

[mec16631-bib-0022] de Voogd, N. J. , Becking, L. E. , & Cleary, D. F. R. (2009). Sponge community composition in the Derawan Islands, NE Kalimantan, Indonesia. Marine Ecology Progress Series, 396, 219–230. 10.3354/meps08349

[mec16631-bib-0023] de Voogd, N. J. , Cleary, D. F. R. , Hoeksema, B. W. , Noor, A. , & van Soest, R. W. M. (2006). Sponge beta diversity in the Spermonde Archipelago, Indonesia. Marine Ecology Progress Series, 309, 131–142.

[mec16631-bib-0024] de Voogd, N. J. , Cleary, D. F. R. , Polónia, A. R. , & Gomes, N. C. M. (2015). Bacterial community composition and predicted functional ecology of sponges, sediment and seawater from the thousand islands reef complex, West Java, Indonesia. FEMS Microbiology Ecology, 91, fiv019. 10.1093/femsec/fiv019 25764467

[mec16631-bib-0025] de Voogd, N. J. , Gauvin‐Bialecki, A. , Polónia, A. R. M. , & Cleary, D. F. R. (2019). Assessing the bacterial communities of sponges inhabiting the remote western Indian Ocean Island of Mayotte. Marine Ecology, 39(6), e12517. 10.1007/s10482-020-01503-5 33369710

[mec16631-bib-0026] Díez‐Vives, C. , Taboada, S. , Leiva, C. , Busch, K. , Hentschel, U. , & Riesgo, A. (2020). On the way to specificity – Microbiome reflects sponge genetic cluster primarily in highly structured populations. Molecular Ecology, 29(22), 4412–4427. 10.1111/mec.15635 32931063 PMC7756592

[mec16631-bib-0027] Dray, S. , Bauman, D. , Blanchet, G. , Borcard, D. , Clappe, S. , Guenard, G. , Jombart, T. , Larocque, G. , Legendre, P. , Madi, N. , & Wagner, H. H (2020). adespatial: Multivariate Multiscale Spatial Analysis. R package version 0.3‐8. https://CRAN.R‐project.org/package=adespatial.

[mec16631-bib-0028] Ellingsen, K. E. (2002). Soft‐sediment benthic biodiversity on the continental shelf in relation to environmental variability. Marine Ecology Progress Series, 232, 15–27.

[mec16631-bib-0029] Ferreira, M. R. S. , Cleary, D. F. R. , Coelho, F. J. R. C. , Gomes, N. C. M. , Huang, Y. M. , Polónia, A. R. M. , & de Voogd, N. J. (2020). Geographical location and habitat predict variation in prokaryotic community composition of *Suberites diversicolor* . Annals of Microbiology, 70(1), 1–12. 10.1186/s13213-020-01546-z

[mec16631-bib-0030] Gao, Q. , Yang, Y. , Feng, J. , Tian, R. , Guo, X. , Ning, D. , Hale, L. , Wang, M. , Cheng, J. , Wu, L. , Zhao, M. , Zhao, J. , Wu, L. , Qin, Y. , Qi, Q. , Liang, Y. , Sun, B. , Chu, H. , & Zhou, J. (2019). The spatial scale dependence of diazotrophic and bacterial community assembly in paddy soil. Global Ecology and Biogeography, 28(8), 1093–1105. 10.1111/geb.12917

[mec16631-bib-0031] Gilbert, B. , & Lechowicz, M. J. (2004). Neutrality, niches, and dispersal in a temperate forest understory. Proceedings of the National Academy of Sciences of the United States of America, 101(20), 7651–7656. 15128948 10.1073/pnas.0400814101 PMC419661

[mec16631-bib-0032] Godfray, H. C. J. , & Lawton, J. H. (2001). Scale and species numbers. Trends in Ecology & Evolution, 16(7), 400–404. 11403873 10.1016/s0169-5347(01)02150-4

[mec16631-bib-0033] Green, J. L. , Holmes, A. J. , Westoby, M. , Oliver, I. , Briscoe, D. , Dangerfield, M. , Gillings, M. , & Beattie, A. J. (2004). Spatial scaling of microbial eukaryote diversity. Nature, 432(7018), 747–750. 15592411 10.1038/nature03034

[mec16631-bib-0034] Griffiths, S. M. , Antwis, R. E. , Lenzi, L. , Lucaci, A. , Behringer, D. C. , Butler, M. J., IV , & Preziosi, R. F. (2019). Host genetics and geography influence microbiome composition in the sponge *Ircinia campana* . Journal of Animal Ecology, 88(11), 1684–1695. 31325164 10.1111/1365-2656.13065 PMC6899969

[mec16631-bib-0035] Guélat, J. , Jaquiéry, J. , Berset‐Brändli, L. , Pellegrini, E. , Moresi, R. , Broquet, T. , Hirzel, A. H. , & Perrin, N. (2008). Mass effects mediate coexistence in competing shrews. Ecology, 89(7), 2033–2042. 10.1890/07-0905.1 18705388

[mec16631-bib-0036] Hadas, E. , Marie, D. , Shpigel, M. , & Ilan, M. (2006). Virus predation by sponges is a new nutrient‐flow pathway in coral reef food webs. Limnology and Oceanography, 51(3), 1548–1550.

[mec16631-bib-0037] Hamming, R. W. (1950). Error detecting and error correcting codes. The Bell System Technical Journal, 29(2), 147–160.

[mec16631-bib-0038] Hanson, C. A. , Fuhrman, J. A. , Horner‐Devine, M. C. , & Martiny, J. B. (2012). Beyond biogeographic patterns: processes shaping the microbial landscape. Nature Reviews Microbiology, 10(7), 497–506. 10.1038/nrmicro2795 22580365

[mec16631-bib-0039] Harte, J. , McCarthy, S. , Taylor, K. , Kinzig, A. , & Fischer, M. L. (1999). Estimating species‐area relationships from plot to landscape scale using species spatial‐turnover data. Oikos, 86, 45–54.

[mec16631-bib-0040] Hentschel, U. , Fieseler, L. , Wehrl, M. , Gernert, C. , Steinert, M. , Hacker, J. , & Horn, M. (2003). Microbial diversity of marine sponges. In W. E. G. Müller (Eds.), Sponges (Porifera). Progress in molecular and subcellular biology (vol. 37, pp. 59–88). Springer, Berlin, Heidelberg. 10.1007/978-3-642-55519-0_3 15825640

[mec16631-bib-0041] Hentschel, U. , Hopke, J. , Horn, M. , Friedrich, A. B. , Wagner, M. , Hacker, J. , & Moore, B. S. (2002). Molecular evidence for a uniform microbial community in sponges from different oceans. Applied and Environmental Microbiology, 68(9), 4431–4440. 12200297 10.1128/AEM.68.9.4431-4440.2002 PMC124103

[mec16631-bib-0042] Hill, M. , Hill, A. , Lopez, N. , & Harriott, O. (2006). Sponge‐specific bacterial symbionts in the Caribbean sponge, *Chondrilla nucula* (Demospongiae, Chondrosida). Marine Biology, 148(6), 1221–1230.

[mec16631-bib-0043] Hillebrand, H. , Watermann, F. , Karez, R. , & Berninger, U. G. (2001). Differences in species richness patterns between unicellular and multicellular organisms. Oecologia, 126(1), 114–124. 28547430 10.1007/s004420000492

[mec16631-bib-0044] Horner‐Devine, M. C. , Lage, M. , Hughes, J. B. , & Bohannan, B. J. (2004). A taxa–area relationship for bacteria. Nature, 432(7018), 750–753. 15592412 10.1038/nature03073

[mec16631-bib-0045] Hou, J. , Wu, L. , Liu, W. , Ge, Y. , Mu, T. , Zhou, T. , Li, Z. , Zhou, J. , Sun, X. , Luo, Y. , & Christie, P. (2020). Biogeography and diversity patterns of abundant and rare bacterial communities in rice paddy soils across China. Science of the Total Environment, 730, 139116. 10.1016/j.scitotenv.2020.139116 32402971

[mec16631-bib-0046] Hubbell, S. P. (2001). The unified neutral theory of biodiversity and biogeography (MPB‐32) (Vol. 32). Princeton University Press.

[mec16631-bib-0047] Hui, C. , & McGeoch, M. A. (2014). Zeta diversity as a concept and metric that unifies incidence‐based biodiversity patterns. American Naturalist, 184(5), 684–694. 10.1086/678125 25325751

[mec16631-bib-0048] Huq, A. , West, P. A. , Small, E. B. , Huq, M. I. , & Colwell, R. R. (1984). Influence of water temperature, salinity, and pH on survival and growth of toxigenic *Vibrio cholerae* serovar 01 associated with live copepods in laboratory microcosms. Applied and Environmental Microbiology, 48(2), 420–424. 6486784 10.1128/aem.48.2.420-424.1984 PMC241529

[mec16631-bib-0049] Johnson, C. N. , Bowers, J. C. , Griffitt, K. J. , Molina, V. , Clostio, R. W. , Pei, S. , Laws, E. , Paranjpye, R. N. , Strom, M. S. , Chen, A. , Hasan, N. A. , Huq, A. , Noriea, N. F., 3rd , Grimes, D. J. , & Colwell, R. R. (2012). Ecology of *Vibrio parahaemolyticus* and *Vibrio vulnificus* in the coastal and estuarine waters of Louisiana, Maryland, Mississippi, and Washington (United States). Applied and Environmental Microbiology, 78(20), 7249–7257. 10.1128/AEM.01296-12 22865080 PMC3457101

[mec16631-bib-0050] Jones, M. M. , Tuomisto, H. , Clark, D. B. , & Olivas, P. (2006). Effects of mesoscale environmental heterogeneity and dispersal limitation on floristic variation in rain forest ferns. Journal of Ecology, 94, 181–195.

[mec16631-bib-0051] Julie, D. , Solen, L. , Antoine, V. , Annick, D. , & Dominique, H. H. (2010). Ecology of pathogenic and non‐pathogenic *Vibrio parahaemolyticus* on the French Atlantic coast. Effects of temperature, salinity, turbidity and chlorophyll a. Environmental Microbiology, 12(4), 929–937. 20100246 10.1111/j.1462-2920.2009.02136.x

[mec16631-bib-0052] Klindworth, A. , Pruesse, E. , Schweer, T. , Peplies, J. , Quast, C. , Horn, M. , & Glöckner, F. O. (2013). Evaluation of general 16S ribosomal RNA gene PCR primers for classical and next‐generation sequencing‐based diversity studies. Nucleic Acids Research, 41(1), e1. 10.1093/nar/gks808 22933715 PMC3592464

[mec16631-bib-0053] Kowalke, J. (2000). Ecology and energetics of two Antarctic sponges. Journal of Experimental Marine Biology and Ecology, 247(1), 85–97. 10727689 10.1016/s0022-0981(00)00141-6

[mec16631-bib-0054] Landesman, W. J. , Nelson, D. M. , & Fitzpatrick, M. C. (2014). Soil properties and tree species drive ß‐diversity of soil bacterial communities. Soil Biology and Biochemistry, 76, 201–209.

[mec16631-bib-0055] Lear, G. , Bellamy, J. , Case, B. S. , Lee, J. E. , & Buckley, H. L. (2014). Fine‐scale spatial patterns in bacterial community composition and function within freshwater ponds. The ISME Journal, 8(8), 1715–1726. 10.1038/ismej.2014.21 24577354 PMC4817609

[mec16631-bib-0056] Legendre, P. , Borcard, D. , & Peres‐Neto, P. R. (2005). Analyzing beta diversity: Partitioning the spatial variation of community composition data. Ecological Monographs, 75(4), 435–450.

[mec16631-bib-0057] Legendre, P. , & Gallagher, E. D. (2001). Ecologically meaningful transformations for ordination of species data. Oecologia, 129(2), 271–280. 28547606 10.1007/s004420100716

[mec16631-bib-0058] Leibold, M. A. , Holyoak, M. , Mouquet, N. , Amarasekare, P. , Chase, J. M. , Hoopes, M. F. , Holt, R. D. , Shurin, J. B. , Law, R. , Tilman, D. , Loreau, M. , & Gonzalez, A. (2004). The metacommunity concept: A framework for multi‐scale community ecology. Ecology Letters, 7(7), 601–613.

[mec16631-bib-0059] Lichstein, J. W. (2007). Multiple regression on distance matrices: A multivariate spatial analysis tool. Plant Ecology, 188(2), 117–131.

[mec16631-bib-0060] Locey, K. J. , Muscarella, M. E. , Larsen, M. L. , Bray, S. R. , Jones, S. E. , & Lennon, J. T. (2020). Dormancy dampens the microbial distance–decay relationship. Philosophical Transactions of the Royal Society B, 375(1798), 20190243. 10.1098/rstb.2019.0243 PMC7133528 32200741

[mec16631-bib-0061] Maas, D. L. , Prost, S. , Bi, K. , Smith, L. L. , Armstrong, E. E. , Aji, L. P. , Toha, A. H. A. , Gillespie, R. G. , & Becking, L. E. (2018). Rapid divergence of mussel populations despite incomplete barriers to dispersal. Molecular Ecology, 27(7), 1556–1571. 10.1111/mec.14556 29575349

[mec16631-bib-0062] Makarenkov, V. , & Legendre, P. (2002). Nonlinear redundancy analysis and canonical correspondence analysis based on polynomial regression. Ecology, 83(4), 1146–1161. 10.1890/0012-9658(2002)083[1146:nraacc]2.0.co;2

[mec16631-bib-0063] Marino, C. M. , Pawlik, J. R. , López‐Legentil, S. , & Erwin, P. M. (2017). Latitudinal variation in the microbiome of the sponge *Ircinia campana* correlates with host haplotype but not anti‐predatory chemical defense. Marine Ecology Progress Series, 565, 53–66.

[mec16631-bib-0064] Martiny, J. B. , Eisen, J. A. , Penn, K. , Allison, S. D. , & Horner‐Devine, M. C. (2011). Drivers of bacterial β‐diversity depend on spatial scale. Proceedings of the National Academy of Sciences of the United States of America, 108(19), 7850–7854. 21518859 10.1073/pnas.1016308108 PMC3093525

[mec16631-bib-0065] Martiny, J. B. H. , Bohannan, B. J. , Brown, J. H. , Colwell, R. K. , Fuhrman, J. A. , Green, J. L. , Horner‐Devine, M. C. , Kane, M. , Krumins, J. A. , Kuske, C. R. , Morin, P. J. , Naeem, S. , Ovreås, L. , Reysenbach, A. L. , Smith, V. H. , & Staley, J. T. (2006). Microbial biogeography: Putting microorganisms on the map. Nature Reviews Microbiology, 4(2), 102–112. 16415926 10.1038/nrmicro1341

[mec16631-bib-0066] Meyer, K. M. , Memiaghe, H. , Korte, L. , Kenfack, D. , Alonso, A. , & Bohannan, B. J. (2018). Why do microbes exhibit weak biogeographic patterns? The ISME Journal, 12(6), 1404–1413. 10.1038/s41396-018-0103-3 29662146 PMC5956095

[mec16631-bib-0067] Moitinho‐Silva, L. , Seridi, L. , Ryu, T. , Voolstra, C. R. , Ravasi, T. , & Hentschel, U. (2014). Revealing microbial functional activities in the Red Sea sponge *Stylissa carteri* by metatranscriptomics. Environmental Microbiology, 16(12), 3683–3698. 24920529 10.1111/1462-2920.12533

[mec16631-bib-0068] Moitinho‐Silva, L. , Steinert, G. , Nielsen, S. , Hardoim, C. C. , Wu, Y. C. , McCormack, G. P. , López‐Legentil, S. , Marchant, R. , Webster, N. , Thomas, T. , & Hentschel, U. (2017). Predicting the HMA‐LMA status in marine sponges by machine learning. Frontiers in Microbiology, 8, 752. 28533766 10.3389/fmicb.2017.00752 PMC5421222

[mec16631-bib-0069] Morlon, H. , Chuyong, G. , Condit, R. , Hubbell, S. , Kenfack, D. , Thomas, D. , Valencia, R. , & Green, J. L. (2008). A general framework for the distance–decay of similarity in ecological communities. Ecology Letters, 11(9), 904–917. 10.1111/j.1461-0248.2008.01202.x 18494792 PMC2613237

[mec16631-bib-0070] Nekola, J. C. , & White, P. S. (1999). The distance decay of similarity in biogeography and ecology. Journal of Biogeography, 26(4), 867–878.

[mec16631-bib-0071] Papke, R. T. , Ramsing, N. B. , Bateson, M. M. , & Ward, D. M. (2003). Geographical isolation in hot spring cyanobacteria. Environmental Microbiology, 5(8), 650–659. 12871232 10.1046/j.1462-2920.2003.00460.x

[mec16631-bib-0072] Pile, A. J. , Patterson, M. R. , & Witman, J. D. (1996). In situ grazing on plankton <10 μm by the boreal sponge *Mycale lingua* . Marine Ecology Progress Series, 141, 95–102.

[mec16631-bib-0073] Pita, L. , Turon, X. , López‐Legentil, S. , & Erwin, P. M. (2013). Host rules: Spatial stability of bacterial communities associated with marine sponges (*Ircinia* spp.) in the Western Mediterranean Sea. FEMS Microbiology Ecology, 86(2), 268–276. 10.1111/1574-6941.12159 23837533

[mec16631-bib-0074] Polónia, A. R. M. , Cleary, D. F. R. , de Voogd, N. J. , Renema, W. , Hoeksema, B. W. , Martins, A. , & Gomes, N. C. M. (2015). Habitat and water quality variables as predictors of community composition in an Indonesian coral reef: A multi‐taxon study in the Spermonde Archipelago. Science of the Total Environment, 537, 139–151. 10.1016/j.scitotenv.2015.07.102 26282748

[mec16631-bib-0075] Polónia, A. R. M. , Cleary, D. F. R. , Duarte, L. N. , de Voogd, N. J. , & Gomes, N. C. M. (2014). Composition of Archaea in seawater, sediment, and sponges in the Kepulauan Seribu reef system, Indonesia. Microbial Ecology, 67(3), 553–567. 24477923 10.1007/s00248-013-0365-2

[mec16631-bib-0076] Polónia, A. R. M. , Cleary, D. F. R. , Freitas, R. , de Voogd, N. J. , & Gomes, N. C. M. (2015). The putative functional ecology and distribution of archaeal communities in sponges, sediment and seawater in a coral reef environment. Molecular Ecology, 24(2), 409–423. 25438824 10.1111/mec.13024

[mec16631-bib-0077] Polónia, A. R. M. , Cleary, D. F. R. , Freitas, R. , Gomes, N. C. M. , & de Voogd, N. J. (2017). Archaeal and bacterial communities of *Xestospongia testudinaria* and sediment differ in diversity, composition and predicted function in an Indonesian coral reef environment. Journal of Sea Research, 119, 37–53.

[mec16631-bib-0078] Preston, F. W. (1960). Time and space and the variation of species. Ecology, 41(4), 612–627.

[mec16631-bib-0079] R Core Team (2022). R: A Language and Environment for Statistical Computing. https://www.R‐project.org.

[mec16631-bib-0080] Reiswig, H. M. (1971). Particle feeding in natural populations of three marine demosponges. The Biological Bulletin, 141(3), 568–591.

[mec16631-bib-0081] Reiswig, H. M. (1975). Bacteria as food for temperate‐water marine sponges. Canadian Journal of Zoology, 53(5), 582–589.

[mec16631-bib-0082] Reiswig, H. M. (1981). Partial carbon and energy budgets of the bacteriosponge *Verongia fistularis* (Porifera: Demospongiae) in Barbados. Marine Ecology, 2(4), 273–293. 10.1111/j.1439-0485.1981.tb00271.x

[mec16631-bib-0083] Reiswig, H. M. (1990). In situ feeding in 2 shallow‐water hexactinellid sponges. In K. Rützler (Ed.), New perspectives in sponge biology (pp. 504–510). Smithsonian Institution Press.

[mec16631-bib-0084] Reveillaud, J. , Maignien, L. , Eren, A. M. , Huber, J. A. , Apprill, A. , Sogin, M. L. , & Vanreusel, A. (2014). Host‐specificity among abundant and rare taxa in the sponge microbiome. The ISME Journal, 8(6), 1198–1209. 24401862 10.1038/ismej.2013.227 PMC4030224

[mec16631-bib-0085] Ribes, M. , Coma, R. , & Gili, J. M. (1999). Natural diet and grazing rate of the temperate sponge *Dysidea avara* (Demospongiae, Dendroceratida) throughout an annual cycle. Marine Ecology Progress Series, 176, 179–190.

[mec16631-bib-0086] Rosenzweig, M. L. (1995). Species diversity in space and time. Cambridge University Press.

[mec16631-bib-0087] Schliep, K. P. (2011). phangorn: Phylogenetic analysis in R. Bioinformatics, 27(4), 592–593. 21169378 10.1093/bioinformatics/btq706 PMC3035803

[mec16631-bib-0088] Spencer, M. , Schwartz, S. S. , & Blaustein, L. (2002). Are there fine‐scale spatial patterns in community similarity among temporary freshwater pools? Global Ecology and Biogeography, 11, 71–78.

[mec16631-bib-0089] Stauder, M. , Vezzulli, L. , Pezzati, E. , Repetto, B. , & Pruzzo, C. (2010). Temperature affects *Vibrio cholerae* O1 El Tor persistence in the aquatic environment via an enhanced expression of GbpA and MSHA adhesins. Environmental Microbiology Reports, 2(1), 140–144. 23766009 10.1111/j.1758-2229.2009.00121.x

[mec16631-bib-0090] Sun, D. , Chen, Y. , Feng, Y. , Liu, Z. , Peng, X. , Cai, Y. , Yu, P. , & Wang, C. (2021). Seasonal variation in size diversity: Explaining the spatial mismatch between phytoplankton and mesozooplankton in fishing grounds of the East China Sea. Ecological Indicators, 131, 108201.

[mec16631-bib-0091] Swierts, T. , Cleary, D. F. R. , & de Voogd, N. J. (2018). Biogeography of prokaryote communities in closely related giant barrel sponges across the Indo‐Pacific. FEMS Microbiology Ecology, 94(12), fiy194. 10.1093/femsec/fiy194 30289448 PMC6196991

[mec16631-bib-0092] Swierts, T. , Peijnenburg, K. T. C. A. , de Leeuw, C. , Cleary, D. F. , Hörnlein, C. , Setiawan, E. , Wörheide, G. , Erpenbeck, D. , & de Voogd, N. J. (2013). Lock, stock and two different barrels: Morphological and genetic variation of the Indo‐Pacific sponge *Xestospongia testudinaria* around Lembeh Island. PLoS One, 8, e74396. 10.1371/journal.pone.0074396 24069308 PMC3771914

[mec16631-bib-0093] Swierts, T. , Peijnenburg, K. T. C. A. , de Leeuw, C. A. , Breeuwer, J. A. , Cleary, D. F. R. , & de Voogd, N. J. (2017). Globally intertwined evolutionary history of giant barrel sponges. Coral Reefs, 36(3), 933–945. 10.1007/s00338-017-1585-6

[mec16631-bib-0094] Tan, K. S. , Acerbi, E. , & Lauro, F. M. (2016). Marine habitats and biodiversity of Singapore's coastal waters: A review. Regional Studies in Marine Science, 8, 340–352. 10.1016/j.rsma.2016.01.008

[mec16631-bib-0095] Taylor, M. W. , Radax, R. , Steger, D. , & Wagner, M. (2007). Sponge‐associated microorganisms: Evolution, ecology, and biotechnological potential. Microbiology and Molecular Biology Reviews, 71(2), 295–347. 17554047 10.1128/MMBR.00040-06 PMC1899876

[mec16631-bib-0096] Taylor, M. W. , Schupp, P. J. , De Nys, R. , Kjelleberg, S. , & Steinberg, P. D. (2005). Biogeography of bacteria associated with the marine sponge *Cymbastela concentrica* . Environmental Microbiology, 7(3), 419–433. 15683402 10.1111/j.1462-2920.2004.00711.x

[mec16631-bib-0097] Thomas, T. , Moitinho‐Silva, L. , Lurgi, M. , Björk, J. R. , Easson, C. , Astudillo‐García, C. , Olson, J. B. , Erwin, P. M. , López‐Legentil, S. , Luter, H. , Chaves‐Fonnegra, A. , Costa, R. , Schupp, P. J. , Steindler, L. , Erpenbeck, D. , Gilbert, J. , Knight, R. , Ackermann, G. , Victor Lopez, J. , … Webster, N. S. (2016). Diversity, structure and convergent evolution of the global sponge microbiome. Nature Communications, 7(1), 11870. 10.1038/ncomms11870 PMC4912640 27306690

[mec16631-bib-0098] Thompson, J. R. , Rivera, H. E. , Closek, C. J. , & Medina, M. (2015). Microbes in the coral holobiont: Partners through evolution, development, and ecological interactions. Frontiers in Cellular and Infection Microbiology, 4, 176. 10.3389/fcimb.2014.00176 25621279 PMC4286716

[mec16631-bib-0099] Torgo, L. (2016). Data mining with R: Learning with case studies. Chapman and Hall/CRC. http://www.dcc.fc.up.pt/~ltorgo/DataMiningWithR

[mec16631-bib-0100] Tuomisto, H. , & Ruokolainen, K. (2006). Analyzing or explaining beta diversity? Understanding the targets of different methods of analysis. Ecology, 87(11), 2697–2708. 10.1890/0012-9658 (2006). 17168014

[mec16631-bib-0101] Tuomisto, H. , Ruokolainen, K. , & Yli‐Halla, M. (2003). Dispersal, environment, and floristic variation of western Amazonian forests. Science, 299(5604), 241–244. 10.1126/science.1078037 12522248

[mec16631-bib-0102] Vacelet, J. , & Donadey, C. (1977). Electron microscope study of the association between some sponges and bacteria. Journal of Experimental Marine Biology and Ecology, 30(3), 301–314.

[mec16631-bib-0103] Wang, Z. B. , Sun, Y. Y. , Li, Y. , Chen, X. L. , Wang, P. , Ding, H. T. , Chen, B. , Zhang, X. Y. , Song, X. Y. , Wang, M. , McMinn, A. , Zhang, Y. Z. , & Qin, Q. L. (2020). Significant bacterial distance‐decay relationship in continuous, well‐connected Southern Ocean surface water. Microbial Ecology, 80(1), 73–80. 10.1007/s00248-019-01472-x 31863131

[mec16631-bib-0104] Webster, N. S. , Negri, A. P. , Munro, M. M. , & Battershill, C. N. (2004). Diverse microbial communities inhabit Antarctic sponges. Environmental Microbiology, 6(3), 288–300. 14871212 10.1111/j.1462-2920.2004.00570.x

[mec16631-bib-0105] Weisz, J. B. , Lindquist, N. , & Martens, C. S. (2008). Do associated microbial abundances impact marine demosponge pumping rates and tissue densities? Oecologia, 155(2), 367–376. 10.1007/s00442-007-0910-0 18030495

[mec16631-bib-0106] Whitaker, R. J. , Grogan, D. W. , & Taylor, J. W. (2003). Geographic barriers isolate endemic populations of hyperthermophilic archaea. Science, 301(5635), 976–978. 12881573 10.1126/science.1086909

[mec16631-bib-0107] Wilkinson, C. R. (1978). Microbial associations in sponges. I. Ecology, physiology and microbial populations of coral reef sponges. Marine Biology, 49(2), 161–167. 10.1007/bf00387117

[mec16631-bib-0108] Woodcock, S. , Curtis, T. P. , Head, I. M. , Lunn, M. , & Sloan, W. T. (2006). Taxa–area relationships for microbes: The unsampled and the unseen. Ecology Letters, 9(7), 805–812. 16796570 10.1111/j.1461-0248.2006.00929.x

[mec16631-bib-0109] Wu, W. , Lu, H. P. , Sastri, A. , Yeh, Y. C. , Gong, G. C. , Chou, W. C. , & Hsieh, C. H. (2018). Contrasting the relative importance of species sorting and dispersal limitation in shaping marine bacterial versus protist communities. The ISME Journal, 12(2), 485–494. 29125596 10.1038/ismej.2017.183 PMC5776463

